# Explaining the process of learning about dignity by undergraduate nursing students: A grounded theory study

**DOI:** 10.1177/09697330241265409

**Published:** 2024-07-24

**Authors:** Hugo Franco, Sílvia Caldeira, Lucília Nunes

**Affiliations:** 70869 Instituto Politécnico de Setúbal; Universidade Católica Portuguesa; 70869 Instituto Politécnico de Setúbal; Comprehensive Health Research Centre [CHRC]

**Keywords:** Dignity, ethics education, grounded theory, nursing students, qualitative research

## Abstract

**Background:**

The learning process about dignity and how undergraduate nursing students experience and use this ethical knowledge is an under-represented field in nursing research. To overcome the lack of conceptual clarity, it is important to understand what processes and dimensions students develop to support this learning outcome.

**Objective:**

This study aimed to explain the process of learning about dignity by undergraduate nursing students.

**Research design and methods:**

A qualitative study was conducted using the grounded theory method.

**Participants and research context:**

Data was collected through free reports and in-depth semi-structured interviews with 20 participants. A focus group was held for the selective coding. Sampling began purposefully and evolved into theoretical. Reflective and theoretical memos were generated from the data collection and constant comparison. Data analysis was performed using qualitative data analysis software using Corbin and Strauss’ method.

**Ethical considerations:**

The research was approved by a specialized research ethics committee from a Health School.

**Findings:**

The process of learning about dignity by undergraduate nursing students revealed ‘recognition of dignity’ as the core category, supported by five main categories: ‘proto-conscience of dignity’, ‘pathway to nursing’, ‘consciousness of dignity’, ‘ways of learning’, and ‘becoming capable’. These categories illustrate the processes and dimensions involved in nursing students’ concept translation of dignity learning, allowing a theory to emerge.

**Conclusions:**

The ‘Recognition of Dignity’ theory aims to contribute to developing educational, training, and supervision processes for nursing programs. It seeks to enhance the ethical and moral development of undergraduate nursing students by helping them understand the concept of dignity and its fundamental importance in nursing.

## Introduction

Respect for dignity is a fundamental principle for nurses, and dignity is an essential value in nursing care.^1,2^ Recognizing, respecting, enhancing, and preserving dignity in healthcare is a challenge and a need for undergraduate nursing students.^3–6^

Education is a crucial process for human beings as it develops their capacities.^7^ Nursing students experience a period of transformation based on their educational processes. An essential aspect of the academic curriculum is reinforcing students with an ethical ethos, which ensures that ethical values, such as human dignity, are fostered.^8,9^ Knowledge and ethical understanding of dignity in nursing care should be vital to the nursing curriculum.^10^

The learning process about dignity and how students experience and use this ethical knowledge is an under-represented field in nursing research.^10–13^ Using grounded theory methodology, this study aims to explain the process of learning about dignity in undergraduate nursing students.

## Background

The concept of dignity holds significant importance in the knowledge structure of the nursing discipline. The earliest links between dignity and care in nursing science date to 1970 in Wiedenbah’s definition of Nursing Philosophy.^14,15^ The juxtaposition of ethical issues related to the value of dignity and nursing science can lead to future research.^16^ Concepts that were included in a philosophy of care were defined. These concepts included respect for dignity, worth, autonomy and the sense of being an individual.^15^ Subsequently, Watson^17–19^ developed the theory of human care, from which new meanings for dignity in nursing emerged. Respect for dignity is the moral imperative of nursing and a core ethical principle.^15^

Several studies and syntheses on the concept of dignity are available nowadays.^20–29^ Healthcare providers and researchers need to describe the meanings and use of the concept clearly. However, these studies do not explain the process by which students make the connection between the concept and the sensibility for ethical competence.

Some suggestions can be found concerning nursing curricula and professional integration programs for nurses that should prioritize education on dignity, as students reportedly find it easier to understand respecting dignity in clinical placements than the concept of dignity as a theoretical task.^30^ Research into teaching dignity to undergraduate nursing students has shown that the concept of dignity in care can be a challenge to existing health cultures. Students suggest that the curriculum could benefit from a more extensive study of dignity.^10,30^ Nevertheless, how students learn to theorize about dignity in Nursing care is unclear.

Research on education concerning dignity concluded that no conceptual definition of dignity-enhancing care in the UK is available. No consensus lines exist on how dignity learning is carried out and assessed.^11,12^ Because of the intrinsic complexity of dignity, it is difficult to know how dignity can be expressed and how students can gain an understanding of the phenomenon.^6^

As part of other subjects, teaching about dignity in the nursing curriculum requires a careful and nuanced approach to fully appreciate its value and complexity. It is crucial to critically explore the various attributes that make up dignity, both in theory and in practice, to ensure a comprehensive understanding of each attribute.^13^ To overcome this lack of clarity, it would be critical to understand what processes and dimensions the students develop to support this learning outcome. We therefore found it important and necessary to perform this study, which was led by the question: How does the undergraduate nursing student learn about dignity? The goal of this study was to explain the process of learning about dignity by undergraduate nursing students.

## Methods

### Study design and research context

A qualitative study was carried out in Portugal between 2021 and 2023 based on a grounded theory approach, using Corbin and Strauss’s^31^ method, which is aligned to explore students’ experience with the learning process about dignity.

### Data collection and analysis

The study population comprised nursing undergraduate students at a university health centre in southern Portugal. The selection of these students was based on the researchers’ convenience and access to the subjects to facilitate the possibility of repeating the interview with one of the participants or scheduling new observations or contacts. The number of participants was not predetermined; instead, it was the result of a deliberate and theoretical sampling process. In this process, the identification and development of concepts determined the need for the type of information to be collected, as well as the most appropriate instruments and participants throughout the study. The data were collected through the participation of 20 students enrolled in the undergraduate nursing programme, selected randomly during the academic years 2020/2021 and 2021/2022. The sampling strategies employed in Grounded Theory are based on intentional and theoretical sampling, which can be defined as the process of collecting data to develop a theory. The data collection process is guided by the emerging theory.^26^ Theoretical sampling aims to discover categories, their properties, and the relationships between them. In theoretical sampling, it is assumed that theories formulated for one group will also hold true for other groups under similar conditions.^31,32^

The initial recruitment period commenced in 2020/2021 for the first- and fourth-year classes. The study was publicized via email and the higher education institution information channels. Participation was initially free and at the initiative of the student, who was then provided with an invitation letter and a free, informed consent form to participate in the study. In this purposive sampling stage, the experiences of five^5^ first-year undergraduate nursing students and four^4^ fourth-year students were collected.

We then evolved to a theoretical sampling phase during the 2021/2022 school year. A criterion of intentionality and theoretical purpose was considered, and eleven^11^ fourth-year students were selected to participate, whose marks in the specific ethics curricular units throughout the course were between good and excellent. In grounded theory, this theoretical intentionality allowed us to collect the data necessary for axial and selective coding.

Free reports and semi-structured interviews were used in the simple and axial categorization phase, while a focus group was held to formulate the selective coding phase.

The data produced in the free reports followed the script previously drawn up for this purpose. It was constructed according to Walker and Avant’s concept analysis methodology^28^ and sought to generate data on the students’ understanding of dignity about its central attributes, antecedents (conditions influencing dignity) and consequents (social responses to the pursuit of dignity) that the students presented on the concept at the time. The reports were completed individually, with participants being informed that there were no correct or incorrect responses but rather their personal experiences. Participants were permitted to answer the questions in a private setting. The interview script and exploratory approaches were informed by Strauss’s guidelines for tracing participants’ perspectives on definitions, situations, experiences, main concerns, assumptions, implicit meanings, and tacit intuition. The semi-structured interviews were designed to explore the symbolic meaning and social interactions that influence participants’ views on dignity learning. Initially, a set of main topics to be addressed was defined, and the previously established script underwent modification due to the production and comparison of the data. This modification was designed to facilitate the interviewees’ expression of opinions on the subject under study, allowing them to follow a chosen path.^26,29,30^

Given the complexity of the topic under study, a preliminary structure was presented at the outset. Some questions posed in the script are as follows:

*Have you dealt with the issue of dignity at any point in your life? If so, in what context? Can you share that experience in detail?*

*Can you now describe your understanding of dignity in detail? If you had to explain to me what dignity is, how would you do it?*

*Did you take part in any school projects or training on dignity while you were growing up? If so, can you describe these experiences?*

*In your family, was there any experience you can remember in which you needed to reflect on dignity or the dignity of the Other? What was that experience like?*

*In the context of undergraduate nursing course, can you describe how you approached the concept of dignity? Can you give specific examples?*

The focus group was used in the final phase of Grounded Theory, according to the premise of generating discussion of consensus or divergence on the categories of the emerging theory. The aim was to consolidate the selective coding emerging from the semi-structured interviews, presenting the central categories of the theory on dignity learning to the students taking part in the study.

The invitation to the focus group was sent by email following the semi-structured interviews, which were conducted with informed consent forms. Nine students agreed to participate in the focus group. An invitation letter was sent to the participants, which explained the purpose of the focus group. The interview was conducted online on the Microsoft® Teams^33^ platform. The invitation was sent out the day before, after confirming receipt of the consent form for participation. Each participant was assigned an identification code to be used during the focus group when presenting the use of the word. Prior to the commencement of the discussion, the participants were informed that the researcher’s role would be limited to that of a moderator, guiding the group through the script. They were then encouraged to engage in discussion amongst themselves.

The free reports, interviews, and focus group lasted between 45 and 90 min. The interviews and focus group were conducted and recorded in a Microsoft® Teams^33^ private channel. All the information was immediately transcribed into verbatim using the Microsoft® Word^34^(Microsoft® Word for Microsoft 365 MSO, version 2204 Build 15128.20224). From these verbatims, theoretical and reflective memos were created on all the material collected with the support of the MAXQDA 2022 (VERBI software).^35^

### Assessment of data accuracy and stability

To ensure the quality criteria of qualitative research, the assumptions of trustworthiness (credibility, transferability, dependability, and confirmability) from Lincoln and Guba were used.^36^ We also aim to assess quality assumptions regarding the grounded theory method. Birks and Mill’s^37^ criteria (the experience of the researchers, methodological congruence, and process precision) were applied.

The study demonstrated methodological congruence and process precision by using purposive and theoretical sampling, spending extensive time with the data to create analytical, theoretical, and reflective memos, triangulating data collection methods through free reports, interviews, and focus groups, and verifying findings with participants during the focus group phase.

The hypothetical transferability was possible to a certain extent by ensuring an in-depth explanation of the study’s basic context, providing a detailed description in presenting the results and guaranteeing theoretical sufficiency in the analysis of the study’s main categories.

Dependability and confirmability were ensured through peer review by the team and quarterly meetings to analyse and discuss the evolution of the theory. Auditability was achieved using data management software, and a comprehensive process description was provided. All data that could be observed or audited by external bodies was kept secure. To produce the research report, we followed the standards for reporting qualitative research.^38^

### Ethical considerations

The research was approved by a Specialised Research Ethics Committee (Opinion no. 64 A/CC/2021). All students were given information about the study by email and internet channels, which included an invitation letter and free, informed consent that should be handed in written form. Before commencing data collection, the study’s purpose and data collection methodology were explained to participants. After assuring them of the confidentiality of the information, anonymity, and the right to withdraw from the study at any time, students agreed to participate and have the interviews recorded. The quotes used above have been translated into English for publication purposes and are the responsibility of the authors since the original language of expression was Portuguese.

## Findings

The findings were analysed and produced using the constant comparative method, which involved comparing data to identify patterns and themes in a codification process. The data was examined for similarities and differences across all free reports, and semi-structured interviews using the method described by Strauss and Corbin^31^ with the assistance of MAXQDA 2022 (VERBI software).^35^

**Figure 1.**
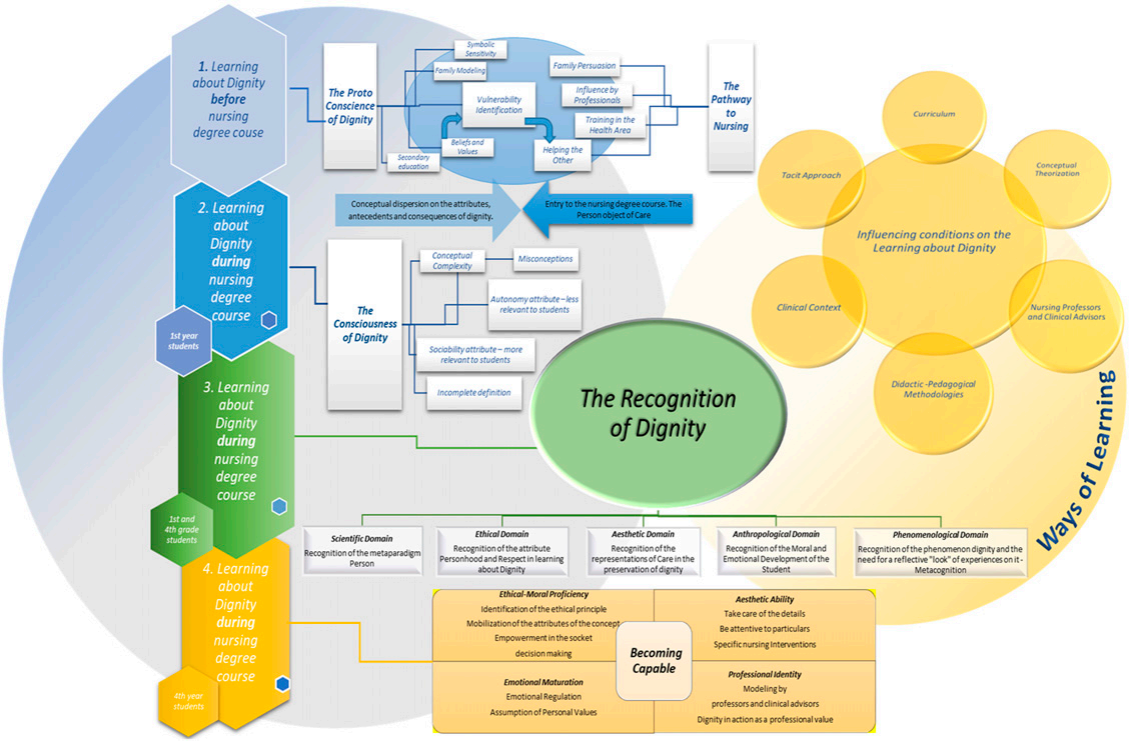
Substantive theory on the recognition of dignity: The process of learning about dignity for nursing students.

Each researcher coded the data independently, and then a consensus was reached. The analysis was conducted at three levels: word by word, line by line, and paragraph by paragraph. This study identified 1200 open codes, classified into six main categories and seventeen subcategories (refer to Table 1). The relationship between the categories obtained from the study is also shown in Figure 1.

**Table 1.** Coding development process.

Open coding aggregation	Axial coding Subcategories	Selective Coding Categories
*Learning about dignity as a child; experiencing situations with family members; ‘finding’ the concept in films, books, the news...; discussing mundane situations with family members; experiencing situations of great fragility in family members/friends; identifying values such as altruism; expression of religious and secondary education.*	Symbolic Sensitivity	***Proto-Conscience of Dignity***
	Family modelling	Properties: learning dignity before the nursing degree course
	Identifying vulnerability	
	Primary and secondary education	
	Beliefs and Values	
*defining the nurse’s role in health care before the program; finding motivation in the family; finding motivation in other nurses for the program; in the relationship with People; contributing to the well-being of Others;*	Family persuasion	***Pathway to Nursing***
	Influence from other nurses	Properties: intersections between dignity and entry to the degree program
	Health training	
	Helping others;	
*Identifying the concept in the curricular units; looking for the attributes of the concept; difficulty in mobilizing the antecedents of the concept.*	Conceptual complexity	***Consciousness of Dignity***
	Misconceptions	Properties: Definition of the concept during the first year of the degree
*Recognizing the Person meta paradigm; the attributes of the concept; the representations of Caring in the preservation of Dignity; the student’s moral and emotional development; and pedagogical strategies.*	Scientific domain	***Recognition of Dignity***
	Ethical domain	Properties: The central process of student learning about dignity
	Aesthetic domain	
	Anthropological domain	
	Phenomenological domain	
*identifying the syllabus; citing the tacit approach; concretizing the conceptual theorizing; reflecting on the clinical contexts; looking at the nursing lecturers and clinical supervisors and naming the effective didactic-pedagogical methodologies.*	Syllabus	***Ways of Learning***
	Tacit approach	Properties: Influencing Conditions on learning Dignity
	Conceptual theorization	
	Clinical contexts	
	Nursing teachers and clinical advisors	
	Didactic-pedagogical methodologies	
*Identifying the ethical principle; mobilizing the attributes of the concept; empowering decision-making; taking care of details; being attentive to details; prescribing Nursing Interventions; developing emotional resilience; assuming personal and professional values; modelling by teachers and clinical advisors; dignity in action as a professional value.*	Ethical-moral proficiency	***Becoming Capable***
	Aesthetic skill	Properties: Results on learning dignity in the 4th year of the degree course
	Emotional maturation	
	Professional identity

Data collection continued until data saturation was reached, which was indicated by no new themes emerging during the analysis. Data saturation was achieved with the participation of twenty^20^ students.

The process of learning about dignity by undergraduate nursing students revealed ‘recognition of dignity’ as the central category, supported by five main categories: ‘proto-conscience of dignity’, ‘pathway to nursing’, ‘consciousness of dignity’, ‘ways of learning’ and ‘becoming capable’.

### The proto-conscience of dignity

The proto-conscience of dignity is defined as a socio-temporal period in the development of the nursing students’ self even before being enrolled in the nursing degree. The understanding of the concept is broad and based on perceptions founded on symbolic sensitivity, family modelling, identification of vulnerability, and construction of beliefs and values. Linked to the biological dimension and the notion of the practical identity of the human being. During this period, students do not attempt to define the concept of dignity. Instead, they gain an understanding of its scope through subjective means, using the abstraction of symbolic sensitivity.

“… when I was younger, I read a book that, thinking about it now, is very much about dignity and at the time, I spoke to my family about it, I can’t remember the name of the book now, but it talks about children being enslaved and sold in Africa, and it’s a true story….” (E7).

The students recognize the intrinsic attribute of dignity because of personal beliefs and values but struggle to identify other dimensions of the concept.

“… I liked going there (in Sunday school), and at the time, I even considered it important, but I’m not a practitioner. I was very young; I started these classes when I was about 6 years old. We were taught that we had to act in favor of others. But at that age, I would never have thought about issues related to dignity or anything like that….” (E6)

At this stage, the identification of vulnerability through family modelling and health/illness experiences emerges, revealing an initial affinity with the social component of the concept of dignity.

“…another situation was when my paternal grandfather died. My grandfather went to the hospital, the emergency department, with a heart attack. I remember being able to go to the emergency room to see my grandfather, and there he was, on a stretcher in the corridor, alone... I looked around... this was the year before I started nursing... I can’t conceive of something like that... He ended up dying alone, on a stretcher, leaning against a corridor wall.... It was very sad.”(E5).

### Pathway to nursing

The path to nursing is often a student choice, sometimes influenced by family members or other health professionals.

“…That’s why I decided to go into nursing. I also have some family and friends who are nurses. They discussed the good things about nursing and the relationship with others.”: “I already have family members in nursing, and it’s always been an area, especially pediatrics, that I’ve been interested in, and I started to look at nursing with different eyes, and I decided.”(E9).

Nevertheless, the personal values of altruism, of being able to help others in situations of vulnerability and fragility, are constantly present:

“This comes from my experience in the scouts and with people, what ignited the flame of nursing was volunteering at the *Telhal* Health Center and seeing those people in such a vulnerable situation and realizing that this was how I wanted to make a difference in people's lives because however little contact we had, which was a weekend contact, it was what I liked to do.” (E5).

### Consciousness of dignity

The consciousness of dignity is defined in a socio-temporal period of the personal nursing students’ development after starting to attend the nursing degree and during the first year of the program (before clinical experience). Subjective perception of the concept, with erroneous or incomplete ideas about it. This awareness translates into the students’ need to define the concept, recognizing the person as the centre of nursing action in the process of ethical-moral and deontological intentions.

“Dignity is a condition that all human beings should be entitled to… Dignity is not something that can be attributed.” (E6).

However, students’ understanding of dignity lacks theoretical and conceptual integration:

“I think it’s quite a difficult concept to define... it even seems to be more on the philosophical side... anyone who approaches it must have a complete literacy and literature, analysing various perspectives and constructing their own... I think that’s it. It’s not really the words, but I'm missing a universal definition... I can’t find it.”(E4).

The first-year students describe only one or two attributes of dignity without relating them to its antecedents and consequents:

“Since dignity is unique and personal, it has to be preserved by everyone, every day, and respect is the key to this. Respecting privacy, the right to choose, everyone’s freedom (of choice, thought, actions, etc...)”(E7).

The conceptions about dignity are flawed and presume complexity, hindering deep reflection on the subject:

“It seems to me to be a difficult concept to grasp, we always have to analyse and understand where we can improve and realise the idea of dignity that we have.” (E1)

“Dignity is something that can be attributed.” (E3).

“By dignifying the person, they may begin to show a superior attitude to others, slack off in maintaining their values and principles and end up taking actions that go against this dignity.” (E4).

### Recognition of dignity

The development of recognition of dignity by students occurs as they progress through the program. It is a transformative process that reorganizes values and becomes a motive, a priori, for their ethical and moral construction:

“Knowing this concept and being alert to this concept has meant that every time I provide care, I pay attention to what this dignity is and what is needed to respect it because perhaps in a first approach, I have all the theory and all the practice that came from the first year, perhaps I wasn't so alert, you know? For example, this question of respecting values and beliefs, maybe we weren’t so alert the first time we saw a person.”(E14).

The recognition of dignity by nursing students includes different domains that help to explain the process of reckoning and learning the concept:

*Ethical* - The importance of recognizing the concepts of dignity, respect, personhood, and autonomy in ethical decision-making is described by students. They also acknowledge the plasticity of these attributes between principle, predictor of moral and ethical action, and value, as represented in the nurse-patient relationship.

“In my opinion, I don’t think we can attribute dignity to anyone since it is a value that is innate to each person; So, I think that as well as dignity being a principle, which is enshrined in our legislation as a maximum principle to be considered, it has even more expression in nursing care; Knowing how to recognize the vulnerability and fragility of others...” (E11);

*Anthropological* -In the anthropological field, students mobilize what we understand to be the ‘mirroring of dignity’. This mirroring involves recognizing their dignity and influencing this notion in their relationship with the ‘other’ within cultural and human-specific elements.

“Dignity can be seen as the recognition/mirroring of positive behaviour/attitudes/actions on the part of a person or a cultural group.” (E12);

*Scientific* – This domain concerns students' theoretical learning about dignity. However, recognition of dignity was not fully conceptualized by the first- and fourth-year students. Students struggled to relate the concept to nursing fundamentals and had difficulty relating it to the nursing episteme on further questioning. With few exceptions, they associated dignity primarily with the metaconcepts of ‘care’ and ‘person’.

“The Person is what dignity has in common and the discipline of nursing for me is the best mirror to reflect this relationship between the two concepts.”(E9);

*Aesthetic* - implies that the importance of detail is acknowledged and that the details in caring for the person can make a difference in favour of the dignity of the ‘other’.

“So, when I bathe, when I make small gestures, even when I talk to the person, although they don’t respond because they can’t, I think it’s about maintaining dignity. Because there it is, the person feels it. They’re not just lying there in bed all day, but they’re interacting as human beings differently. And that would be guaranteeing dignity.” (E6);

*Phenomenological* – Recognizing the phenomenon of dignity and the need for a reflective examination of experiences related to it.

“Yes, I certainly am because the situations we experience during these clinical training courses lead us to reflect, to introspect, even about ourselves in a different way. Some values are being harmonized with other principles. I think that yes, my perception is quite different, even because of people’s life situations, I mean there is almost like a polishing of what is essential for us.” (E17).

### Ways of learning

Participants reported several foundational experiences of learning the concept throughout the course, which define the ‘ways of learning’. This category explains how students perceive the influence of different educational vectors on learning about dignity throughout the course.

The ways of learning are divided into six subcategories:

*Curriculum* – Students recognize several curricular units across the syllabus that play an important role in learning about dignity. This analysis allows them to define the thematic areas within the nursing curriculum where the concept appears most appropriately.

“I think the concept came up more in the ethics, law, and deontology curricular unit, we covered this subject, but I feel from my experience that it has become more consolidated now in the 4th year with the continuing and palliative care classes.”(E17);

*Tacit Approach* – Throughout their degree, students acknowledge that learning about the concept often involves an implicit approach. Teachers and clinical tutors address issues in a theoretical or practical context without mentioning the concept of dignity, which the student later identifies as referring to it.

“And they mentioned in Nursing One, for example, when we talked about needs. That was emphasised. A little implicitly, it led us to think about dignity. But yes. It was always mentioned. I don’t remember if we ever talked about dignity in concrete terms. But I know it was always there.” (E7);

*Conceptual Theorization* – The students identified the theoretical component as one of the key components for learning the concept of dignity. However, no specific conceptual or theoretical framework was identified for teaching about dignity despite the mentioned main attributes.

“But I would explain the definition because there is no concrete definition of the term dignity, but I would try to explain what I knew, what was always talked about throughout the course, and which is important in our relationship with others” (E10);

*Clinical Contexts* – Students often provide examples of learning about dignity in clinical placements. In this subcategory, students explain the antecedents of the concept, such as vulnerability, the clinical environment, person-centred care, and training. However, there appears to be some conceptual scatter about the attributes of the concept:

“And I remember thinking to myself... how am I doing this? how can we... or how can I somehow take more care of the person’s privacy in a context like this where I would sometimes bathe them with the sheet clutched in my hand, which in terms of time ended up taking up more time, and it was more physical than just undressing the person and bathing them. But because it was so confusing for me, I couldn’t do it.”(E15);

*Nursing Lecturers and Clinical Advisors* – When learning about dignity, students consider the training provided by teachers and clinical supervisors to be crucial in facilitating the process of theoretical construction and validating a framework of professional values. This training also helps create a professional identity that aligns with the moral values and deontology of the profession.

“One situation I remember that happened during my last clinical training was a dignity promotion situation in which my nurse supervisor received a terminally ill person. At the time, we were in a pandemic phase in which visitors couldn’t be present. At the time, I remember that the nurse supervisor and the nursing team decided that the person’s family or carer would come in and be with their relative, a little contrary to the rules that were in place at the time in the institution.” (E11);

*Didactic-Pedagogical Methodologies* – During the nursing degree, participants identified methodologies that facilitate learning about dignity. The most effective strategies were analysing and discussing cases, brainstorming concepts, debating, and reflecting on clinical experiences. They mentioned that tutorials, clinical training, and internships were fundamental from a didactic point of view for the phenomenological recognition of dignity:

“In terms of the methodologies used to learn the concept, I remember that in the course on the person in a chronic and palliative situation, we brainstormed concepts and sub-concepts that helped us understand the concept of dignity. Reflecting on the concepts presented and their links with each other was also very important in building a concrete definition of a complex concept.” (E9).

### Becoming capable

Becoming capable is a socio-temporal period of shaping the students’ identity, which culminates with the entry into the final year. It involves processes of competence concerning socio-professional identity. Learning about the concept of dignity maximises the potential for intervention and strengthens the nurse-patient interaction.

This category demonstrates the final impact of learning about dignity and its influence on the student as a social agent and future nursing professional. Becoming Capable encompasses four subcategories:

*Emotional Maturity* – Participants identify emotional maturity as an important factor in becoming capable. By analysing their emotions and emotional resilience in complex contexts, they can develop knowledge about the concept of dignity.

“(...) we realize that running the curtains is much more than that... or pulling the strings is much more than that... and that this has a lot to do with maturity and emotional intelligence, which perhaps in the first year of the course we still don't have...” (E19);

*Ethical and Moral Proficiency* – When learning about dignity, students describe specific situations that make it possible to understand the importance and contribution of their ethical and moral development to understanding the concept of dignity. The principle of dignity and its attributes are mobilized in action through various narratives:

“A person was admitted.... He was in the resuscitation room, and the nurse in charge told me “Look, the doctor ordered blood to be taken for analysis.” And at the time...(pauses) the person was dying. I... I put my hand on his cheek, and he shed a tear, it was a tough moment. I looked at the man’s arm to take the blood and said to my clinical supervisor nurse, “I’m not going to do it. I’m sorry, but I'm not going to. At that moment it was the person I wanted to preserve... not the intervention or the act itself... which had no value whatsoever.” (E18);

*Aesthetic Ability* – The participants learn to preserve dignity through an aesthetic skill developed in clinical contexts. This skill involves attention to detail and developing relationships, which the students say make a difference in maintaining the dignity of the ‘other’.

“The care to close the door, the care to warn the person before we touch, before we turn, before we do anything, any kind of intervention. I think it’s extremely important here to warn the other person, because I’m looking after the other person, I’m touching the other person’s body, the intimacy that isn't mine.” (E16);

*Professional Identity* – The students report instances where professional identity played a crucial role in their development. They learned by example, through the profession’s values, in both positive and negative situations where they observed or experienced the behaviour and intervention of professionals. Learning situations that evoke internal conflicts and feelings about dignity appear to be the most significant in the student’s moral development.

“ I didn’t tell the team, but I don't want to go back and I hope I never have a family member in this service either. On the one hand, it helped me to realize how I don’t want to be and to the point where I thought - I don’t want to be like that, so it ended up influencing my development as a professional, but in the negative, in how I don’t want to be as a professional. It was an experience that left me quite distressed at the time because I didn’t think it should happen. Everyone deserves respect. People shouldn't be treated this way in healthcare.” (E8).

## Discussion

The substantive theory produced made it possible to identify a central phenomenon in learning about dignity according to the student’s experience. This process was called the ‘recognition of dignity’. Axel Honneth^27^ defines recognition as an intersubjective, dialogical, and historical construction through which subjects seek its realization in three essential domains: affection, rights, and social esteem. These domains give rise to self-confidence, self-respect, and self-esteem, respectively. The recognition of dignity is defined as a socio-temporal period in the education of the nursing student’s self after the first year of the nursing course, culminating in the final year of the course. It is integrated, constructivist, and concept-based, assuming affirmation processes in the practice of care. It is built as a conviction and has transformative properties in the student’s personhood. The domains of dignity recognition (ethical, anthropological, scientific, aesthetic, phenomenological) can serve as a theoretical framework for this purpose.

This research has shown that dignity is a crucial concept in nursing and should be given attention in curriculum design and pedagogical strategies students use during their ethical-moral development.^16,39^ It emphasises the necessity for certain complex concepts, such as dignity, to possess an objective conceptual framework that is neither reductive nor impractical to assess pragmatically. Such a framework should prompt nursing students to ‘acknowledge’ a body of knowledge through a transformative process that entails self-reflection.^20^

The research findings also disclose some gaps in students learning about the concept of dignity within the ‘ways of learning’. These gaps are identified in the way that the discipline’s core concepts accompany students’ moral-ethical development. The course descriptions do not explicitly mention the concept of dignity despite it being an essential element of nursing.^13^ This topic’s conceptualization has remained tacit and has not been very operational or linked to disciplinary concepts, despite being mentioned.

Several variables, such as the structure of the curriculum, can influence the outcome despite the individuality inherent in the learning process. The presence of ‘ethics’ courses throughout student’s training to be a nurse provides a theoretical reference to dignity. However, this may not be enough, as students point out the need to link these concepts to other courses, such as mental health, palliative care, in which this ‘recognition of dignity’ seems to emerge due to the condition of vulnerability and stigmatization of the person.

Also, courses related to therapeutic relationships and communication that enable students to develop competencies to manage situations where the dignity of the Other may be or be felt at risk are identified as of value. The internship and clinical placements are considered critical to achieving the ‘Recognition of Dignity’ because they validate the theoretical learning, make it possible to test intervention models, and materialize the definition of the antecedents and consequents of the concept of dignity.

It can be stated that the proposed theory reveals how the metacognitive process of ‘recognition’ is intrinsic to the student but must be provided by the social and educational environment surrounding them. It has been established that despite the students’ ability to describe the elements of (in)dignity in action, their conceptual representation is extremely complex.^4,6,9^ Therefore, it is important for curricula, teachers, and domain theorists to address this challenge.^8,30^

This work has also highlighted that learning to think from a philosophical basis deepens understanding of disciplinary knowledge and should be the essential basis for guiding students’ practice. Philosophical clarity about dignity allows students to assume the distinctions of their practice with a stable foundation in the field of Nursing. It is vital for becoming capable of ethical decision-making.^3,8^

## Strength & limitations

The strengths of this study were that methodological rigour was maintained throughout the research process and that grounded theory as a research product met the criteria of credibility, originality, resonance, and usefulness. It also lies in the original findings provided, completing the knowledge on the process of learning about dignity in this specific population. Using a focus group design, was also an important strategy to increase the confirmability of the research. The findings were deemed transferable to other similar contexts, namely educational institutions, making it possible to focus on the dimensions of learning about the concept, providing categories and attributes that can explain the process of learning about dignity among undergraduate nursing students.

On the other hand, the limitation of all qualitative studies, including the present study, is their dependence on temporal and spatial conditions. Also, constraints relate to participant recruitment in an academic context where the principal investigator and the participants had a teacher-student relationship. This led to the inclusion of respective precautions in the request for an opinion from the ethics committee.

It is important to note that this study was conducted during the global COVID-19 pandemic (2021–22), which affected the data collection process and the students’ teaching and learning experiences related to the phenomenon under study. New challenges, experiences, and emotions arose from this situation, which we have attempted to represent in the way we produced the emerging theory data.

## Conclusion

The process of learning about dignity presents educators with a number of challenges. These include integrating the learning of dignity within the nursing process, while teaching care delivery. There is scope for dignity to be represented in the domain of clinical judgement of undergraduate nursing students and future nurses, with interventions aimed at maintaining or preserving it.

For undergraduate nursing students, the formalization of this knowledge facilitates a series of processes that need to be developed to embed the recognition of dignity. Having this systematized, science-based knowledge, which can support one or more curricular units in the nursing degree, is a strategic element for students to study the ethical and moral dimension.

The formalization of these findings is also innovative for nurses and clinical supervisors, enabling them to make sense of complex learning processes. It makes it possible to use them as a conceptual tool in the context of clinical supervision, whether carried out among peers and/or with students. It encourages reflection and awareness of the care processes they undertake in the context of dignity in care.

The ‘recognition of dignity’ in the actions of future nurses as an ethical foundation and professional value is one of the most significant learning experiences reported by students during this process. The understanding that dignity is the result of mutual recognition. The insight is that dignity is not conferred upon people, but rather it is recognized.

Nursing education may tend to prepare students for their role as nurses based on tasks, skills, and interventions. Students risk becoming masters of the task rather than the discipline, with the consequences this may have for Nursing. Data-based theory highlights how learning about a central and complex concept of the discipline can help develop a referential model for the general care nurse.

In the case of other cultural contexts, it might be useful to replicate the study or discuss the results with a focus group or spark debate about these findings in the context of each cultural reality. However, along with respect for multiculturalism, in the current context of globalization and world affairs, ethical values and human rights tend to stand and remain universal.

The ‘recognition of dignity’ is expected to contribute to the development of educational, training, and supervision processes for nursing students. It aims to enhance their ethical and moral development in understanding the concept of dignity and its fundamental plasticity in the discipline of nursing.
